# Polymeric Hole Transport Materials for Red CsPbI_3_ Perovskite Quantum-Dot Light-Emitting Diodes

**DOI:** 10.3390/polym13060896

**Published:** 2021-03-15

**Authors:** Zong-Liang Tseng, Shih-Hung Lin, Jian-Fu Tang, Yu-Ching Huang, Hsiang-Chih Cheng, Wei-Lun Huang, Yi-Ting Lee, Lung-Chien Chen

**Affiliations:** 1 Department of Electronic Engineering, Ming Chi University of Technology, No. 84, Gungjuan Rd., New Taipei City 24301, Taiwan; a88061446@gmail.com; 2 Department of Electronic Engineering, National Yunlin University of Science and Technology, Yunlin 64002, Taiwan; isshokenmei@yuntech.edu.tw; 3 Bachelor Program in Interdisciplinary Studies, National Yunlin University of Science and Technology, Yunlin 64002, Taiwan; jftang@yuntech.edu.tw; 4 Department of Materials Engineering, Ming Chi University of Technology, No. 84, Gungjuan Rd., New Taipei City 24301, Taiwan; huangyc@mail.mcut.edu.tw; 5 Department of Electro-Optical Engineering, National Taipei University of Technology, 1, Sec. 3, Chung-Hsiao E. Rd., Taipei 10608, Taiwan; kokusoro860127@gmail.com; 6 Center for Organic Photonics and Electronics Research (OPERA), Kyushu University, 744 Motooka, Nishi, Fukuoka 819-0395, Japan; ytlee@opera.kyushu-u.ac.jp

**Keywords:** perovskite, CsPbI_3_, QD, light-emitting diodes

## Abstract

In this study, the performances of red CsPbI_3_-based all-inorganic perovskite quantum-dot light-emitting diodes (IPQLEDs) employing polymeric crystalline Poly(3-hexylthiophene-2,5-diyl) (P3HT), poly(9-vinycarbazole) (PVK), Poly(N,N′-bis-4-butylphenyl-N,N′-bisphenyl)benzidine (Poly-TPD) and 9,9-Bis[4-[(4-ethenylphenyl)methoxy]phenyl]-N2,N7-di-1-naphthalenyl-N2,N7-diphenyl-9H-fluorene-2,7-diamine (VB-FNPD) as the hole transporting layers (HTLs) have been demonstrated. The purpose of this work is an attempt to promote the development of device structures and hole transporting materials for the CsPbI_3_-based IPQLEDs via a comparative study of different HTLs. A full-coverage quantum dot (QD) film without the aggregation can be obtained by coating it with VB-FNPD, and thus, the best external quantum efficiency (EQE) of 7.28% was achieved in the VB-FNPD device. We also reported a standing method to further improve the degree of VB-FNPD polymerization, resulting in the improved device performance, with the EQE of 8.64%.

## 1. Introduction

Organic–inorganic hybrid perovskite APbX_3_ (A is an organic cation, such as CH_3_NH_3_^+^ and NH_2_CH=NH_2_^+^, and X is a halide) has opened new avenues for optoelectronic materials in the recent years [1,2,3,4]. It is well known that CH_3_NH_3_PbX_3_ (MAPbX_3_) and NH_2_CH=NH_2_PbX_3_ (FAPbX_3_) are easily decomposed into PbX_2_ and volatile MAX and FAX in the presence of heat and moisture. To address the issue, more stable all-inorganic perovskite has been developed, such as CsPbX_3_. In 2015, size-controlled and composition-controlled CsPbX_3_ quantum dots (QDs) were first synthesized using a colloidal method [5]. The colloidal CsPbX_3_ QDs exhibit high color purity in photoluminescence (PL) spectrum, and photoluminescence quantum yields (PLQYs) as high as 100%, which makes them candidates for light-emitting diodes (LEDs) [6,7,8,9,10,11,12,13,14,15,16,17,18,19,20,21] and solar cells [22,23,24,25,26,27,28]. Up until now, the development of the colloidal synthesis has been simultaneously promoted in parallel with the performance of CsPbX3 QDs-based optoelectronic devices.

All-inorganic perovskite QD-based LEDs (IPQLEDs) have drawn much attention for their solution-processed fabrication and more flexible application [6,7,8,9,10,11,12,13,14,15,16,17,18,19,20,21]. To improve the IPQLED performance, highly efficient exciton recombination in the QD films is a significantly critical issue. The hole transporting layers (HTLs) reduce the hole injection barrier and block the electron to balance the hole and electron and enhance efficient exciton recombination in the QD films. The poly(9-vinycarbazole) (PVK) was usually used as the HTL in the preliminary stage of the IPQLED development [6,7]. Subsequently, PVK was replaced with Poly(N,N′-bis-4-butylphenyl-N,N′-bisphenyl)benzidine (Poly-TPD), because of improved hole injection efficiency, which could be attributed to the hole mobility of Poly-TPD by about two orders of magnitude higher than that of PVK [8]. To date, Poly-TPD has been selected as the hole transporting material in most of IPQLED studies [12,13,14,15,16,17,18,19,20,21]. Poly(triaryl)amine (PTAA) also shows a high hole mobility, which makes it another good choice for the hole transporting materials. The defect during the formation process of the QD films could be reduced by PTAA [9,10], which is effective in enhancing the radiative recombination. The more crystalline Poly(3-hexylthiophene-2,5-diyl) (P3HT) has a relatively higher hole mobility than that of noncrystalline organic HTLs and is often used as light-harvesting and hole transporting materials for CsPbI_3_-based solar cells [25]. Our previous report demonstrated that a thermal crosslinkable HTL, 9,9-Bis[4-[(4-ethenylphenyl)methoxy]phenyl]-N2,N7-di-1-naphthalenyl-N2,N7-diphenyl-9H-fluorene-2,7-diamine (VB-FNPD), also provides excellent hole mobility and improves the interface between the HTL and the CsPbBr_3_ QD film [11]. On the other hand, the studies of the HTLs for deep red CsPbI_3_-based IPQLEDs are still lacking. Table 1 shows only Poly-TPD and PTAA have been used as the HTLs in the CsPbI_3_-based IPQLEDs. No literature reported the CsPbI_3_-based IPQLEDs using VB-FNPD and P3HT as the HTLs. Therefore, a comparative study of different HTLs for the CsPbI_3_-based IPQLEDs is necessary.

Herein, we studied the performance of CsPbI_3_-based IPQLEDs employing P3HT, PVK, Poly-TPD and VB-FNPD as the HTLs. Meanwhile, a dense and smooth CsPbI_3_ QDs film can be achieved using VB-FNPD HTLs, which are an important factor for the device performance of the IPQLED. We then demonstrated highly bright and efficient CsPbI_3_ IPQLED based on VB-FNPD HTLs, achieving an external quantum efficiency (EQE) of 8.64%. Therefore, we believe that our results may promote the development of device structures and hole transporting materials to achieve stable and low-cost IPQLEDs.

## 2. Experimental Section

### 2.1. Materials

P3HT was purchased from Solarmer (El Monte, CA, USA). Cesium carbonate (Cs_2_CO_3_; 99.995%), octadecene (ODE; 90%), oleic acid (OA; 90%), octylamine (OAm; 90%), hexane (95%), octane (98^+^%), mehyl acetate (99%), PbI_2_ (99.999%), PEDOT:PSS (AI 4083), TPBi and PVK were purchased from Sigma–Aldrich (Munich, Germany). Poly-TPD, n-octylammonium iodide and VB-FNPD were purchased from LUMTEC (Taipei, Taiwan). All the chemicals were used as received.

### 2.2. Synthesis of CsPbI_3_ QDs

Cs_2_CO_3_ (200 mg) was loaded into a 25 mL three-neck flask, along with ODE (9 mL) and OA (0.75 mL), and then stirred and degassed at 120 °C for 30 min under nitrogen flow to obtain a transparent Cs–oleate precursor. The Pb precursor solution was prepared by dissolving 0.09 M of PbI_2_ in 30 mL ODE, 3 mL of OA and 3 mL OAM and then stirring and degassing at 120 °C under nitrogen flow. After PbI_2_ was all dissolved, the temperature was increased to 150 °C, and then a 0.8 mL Cs–oleate precursor was quickly injected into the Pb precursor solution. After 5 s, the reaction was cooled on an ice bath, and red CsPbI_3_ QD crude was obtained. Then n-octylammonium iodide (0.2 mmol) dissolved in toluene (4 mL), as a capping agent was added into the crude. Subsequently, as-prepared crude solution and methyl acetate (16 mL) were centrifugated at 12,000 rpm for 15 min. The precipitate was collected and loaded in 8 mL of hexane and methyl acetate (1:3 v/v), and the solution was centrifuged at 12,000 rpm for 10 min. The precipitate was collected and dispersed in octane (2 mL) and centrifuged for 5 min at 12,000 rpm. Finally, the supernatant was collected and stored at 4 °C.

### 2.3. Device Fabrication

The IPQLEDs were constructed with the architecture of indium tin oxide (ITO)/ PEDOT:PSS (40 nm)/ HTLs (~50 nm)/ CsPbI_3_ QD (~40 nm)/ TPBi (40 nm)/ LiF (1 nm)/Al (100 nm). Here, P3HT, PVK, Poly-TPD and VB-FNPD were used as the HTLs. The patterned ITO substrates were wet-cleaned and then O_2_ Plasma-cleaned. After cleaning, PEDOT:PSS was spin-coated at 8000 rpm for 40 s on the substrate and annealed at 130 °C for 15 min. Then, the samples were loaded to N_2_-filled glove box to deposit HTLs and CsPbI_3_ QDs. All HTLs were spin-coated with a concentration of 4 mg/mL on PEDOT:PSS and then heated at 100 °C for 5 min. The thickness of each HTL was controlled at ~50 nm by adjusting the spinning speed. Before heating, VB-FNPD was held standing still for 0, 20, 40 and 60 min and then heated at 100 °C for 5 min and annealed at 170 °C for 30 min for thermal crosslinking. The CsPbI_3_ QDs were spin-coated with a concentration of 40 mg/mL at 2000 rpm for 60 s. TPBi, LiF and Al cathode were deposited by a thermal evaporation using a shadow mask to define the device area of 2 × 2 mm^2^.

### 2.4. Characterization

Electroluminescence and impedance characteristics were measured through computer-controlled LQ-100R spectrometer (Enlitech, Kaohsiung, Taiwan) and Material Lab XM (SOLARTRON analytical, Leicester, UK), respectively. The absorbance and photoluminescence (PL)/photoluminescence quantum yield (PLQY) were measured using UV-visible spectrophotometer (V-770, JASCO, Tokyo, Japan) in Table 2 and fluorescence spectrophotometer (F-7000, Hitachi, Tokyo, Japan), respectively. The surface roughness was measured using an atomic force microscope (AFM, Bruker, Billerica, MA, USA). The electron microscopy images were obtained by HRTEM (JEM-2100, JEOL, Tokyo, Japan) and FESEM (JSM-7610F, JEOL, Tokyo Japan), respectively.

## 3. Results and Discussion

**Figure 1:**
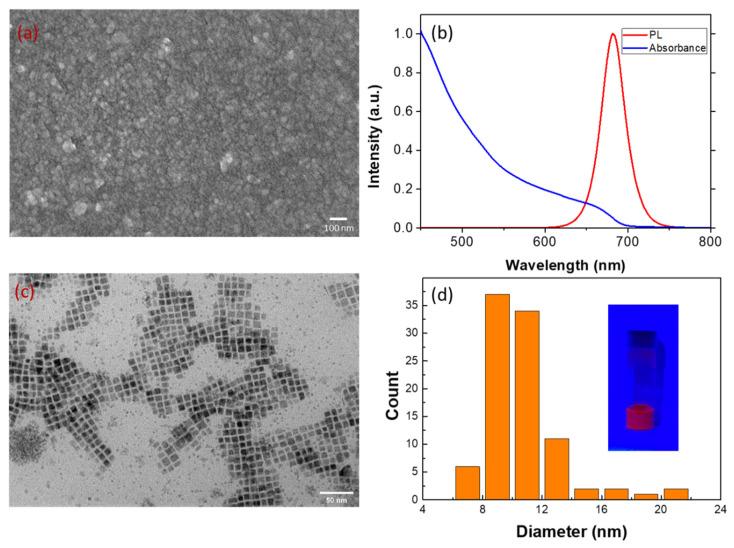
(**a**) SEM image and (**b**) absorbance and photoluminescence (PL) spectra of CsPbI_3_ QDs spun on glass substrates. (**c**) TEM image and (**d**) size distribution of CsPbI_3_ QDs evaluated by (**c**). The inset shows the CsPbI_3_ QDs dispersed in octane and excited under UV light at 365 nm.

Figure 1a shows the planar SEM image of the CsPbI_3_ QD film spun on the glass substrate. Highly dense surface and good crystalline of the CsPbI_3_ QD film can be obtained without obvious aggregations. Such morphology may be attributed to the well-dispersed and high-stability suspensions in the as-synthesized QD dispersions, as shown in the insert in Figure 1. The PL spectrum (Figure 1b) of the CsPbI_3_ QDs film shows a brightly red luminescence at 682 nm with a narrow Full width at half maximum (FWHM) of 35 nm, implying a high color purity and preferred optical property. The absorption edge in the absorption spectrum is close to its emission peak, which agrees with previous reports [22,23,24,25]. TEM image shows as-synthesized CsPbI_3_ QDs are cubic shaped and well-dispersed in octan, with an average size of 10.8 nm (Figure 1c,d). All abovementioned characterization techniques evidently exhibit that the CsPbI_3_ QD dispersion solutions and QD solid films with uniform size and distribution have been successfully obtained.

**Figure 2:**
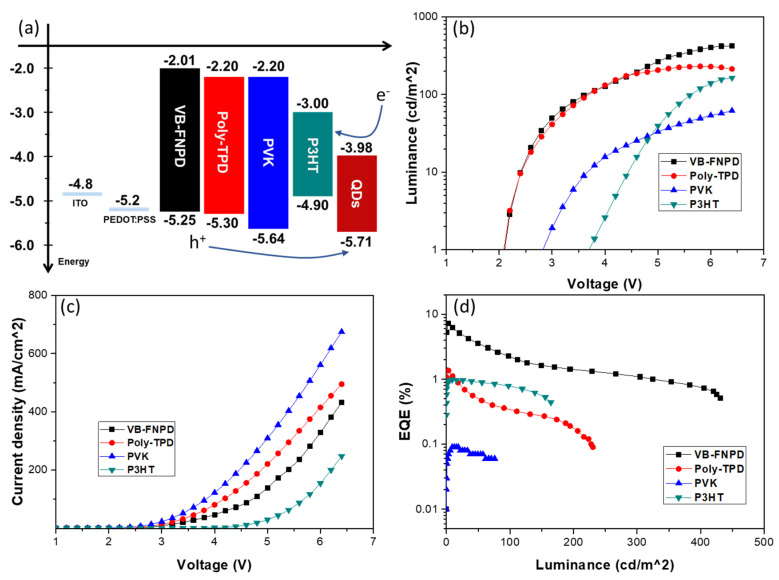
(**a**) Energy levels for different HTLs, (**b**) luminance-voltage (L-V), (**c**) current density-voltage (J-V) and (**d**) external quantum efficiency (EQE) for IPQLED devices with different HTLs.

Figure 2a shows the energy band diagram of the CsPbI_3_ QD layer and each HTL. The highest occupied molecular orbital (HOMO) and lowest unoccupied molecular orbital (LUMO) levels of all layers can be referred to the results in [12,25,29]. Figure 2b–d show the device performance of the CsPbI_3_ IPQLED using different HTLs. LUMO levels of all HTLs are much higher than those of the QD layer, resulting in good electron blocking ability in all HTLs (Figure 2a). HOMO levels of all HTLs are higher than those of the QD layer, indicating that reducing hole injection barrier is preferred to the HTL with the lower HOMO level. Therefore, the tendencies of the current in devices’ different HTLs correspond with the HOMO level of their HTL (Figure 2c). The PVK device shows the highest current, because of the lowest HOMO in PVK, which is agreed with the lowest impedance (Figure S1). In contrast, the lowest current in the P3HT device is caused by the highest HOMO level and hole injection barrier, leading to the highest impedance (Figure S1) and turn-on voltage (biased voltage at 1 cd/m^2^), as shown in Figure 2b. Similar HOMO levels in VB-FNPD and Poly-TPD lead to their same turn-on voltages, but the excellent radiative recombination efficiency in the VB-FNPD device gives it higher EQE. In addition, the PVK device has the highest current, but it simultaneously shows the lowest EQE (Figure 2d), which may be caused by inefficient radiative recombination in the QD layers, leading to its higher turn-on voltage than that of VB-FNPD and Poly-TPD devices (Figure 2b). It is interesting to know what dominates as the carrier recombination efficiency for each device.

**Figure 3:**
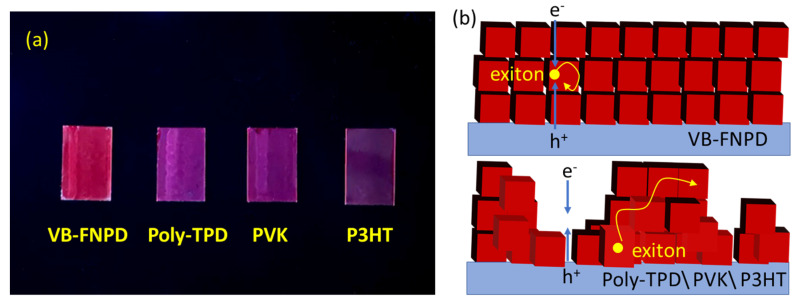
(**a**) CsPbI3 QDs films deposited on 9,9-Bis[4-[(4-ethenylphenyl)methoxy]phenyl]-N2,N7-di-1-naphthalenyl-N2,N7-diphenyl-9H-fluorene-2,7-diamine (VB-FNPD), Poly(N,N′-bis-4-butylphenyl-N,N′-bisphenyl)benzidine (Poly-TPD), poly(9-vinycarbazole) (PVK) and crystalline Poly(3-hexylthiophene-2,5-diyl) (P3HT) and excited under UV light at 365 nm. (**b**) The illustration of the QD aggregations on different HTLs.

In fact, the determination of the carrier recombination efficiency can be easily observed by the naked eye. Figure 3a shows the photograph of CsPbI_3_ QDs films spun on each HTL, in which the VB-FNPD film shows brighter than others. The thicknesses of all QDs films were around 40 nm, measured by Alpha–Step. Hence, the brightness of the QD solids should be attributed to the degree of the aggregation on the different HTL surfaces, rather than the film thickness. When the well-organized array of the colloidal CsPbI_3_ QDs is formed on the surface of the VB-FNPD films without the QD aggregations, the light-induced exciton is limited in a QD nanoparticle to increase the quantum confinement effect, resulting in the improved radiative recombination, as illustrated in Figure 3b. In contrast, the light-induced exciton can transport between nanoparticles, due to the QD aggregations, leading to the increased dissociation possibility of the exciton prior to its radiative decay [30,31]. It is the reason why the brightness of VB-FNPD film is much stronger than that of other films, which is in good agreement with the device results (Figure 2d). The summary of PLQYs for CsPbI_3_ QDs layers spun on different HTLs are listed in Table 1. The PLQY of CsPbI_3_ QDs layer on the glass is higher than the PLQY of those spun on each HTL, which may be because the exciton dissociation is suppressed at the insulated glass [32]. On the other hand, the full-coverage QD films confirm the carrier combination. The films with the QD aggregations provide a leakage path, which is the reason that the current in the Poly-TPD device is higher than that in the VB-FNPD device (Figure 2c). The best performance of 7.28% was achieved in the VB-FNPD device.

**Figure 4:**
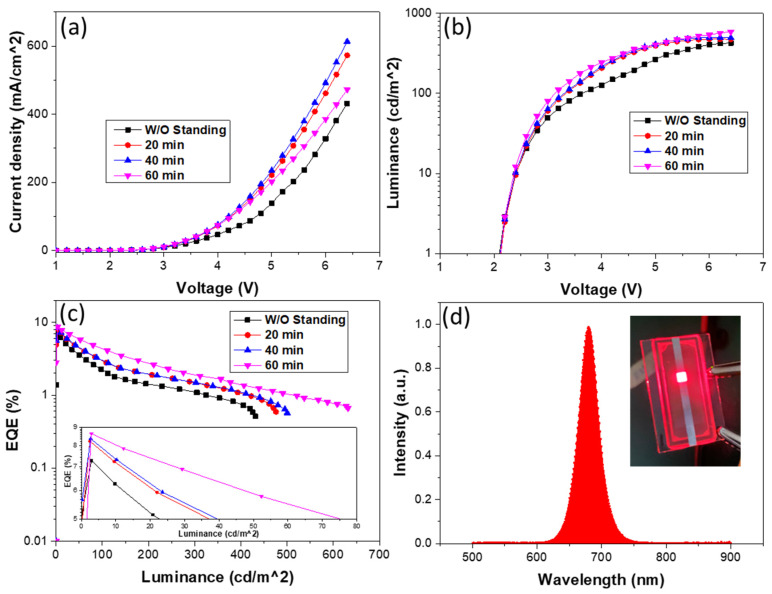
(**a**) Luminance-voltage (L-V), (**b**) current density-voltage (J-V), (**c**) EQE for IPQLED devices based on VB-FNPD HTLs, prepared by different standing times (inset is the enlarged high-efficiency region). (**d**) Electroluminescence (EL) spectrum of the champion device biased at 3 V. Inset is a photograph of the working device with red emission.

**Figure 5:**
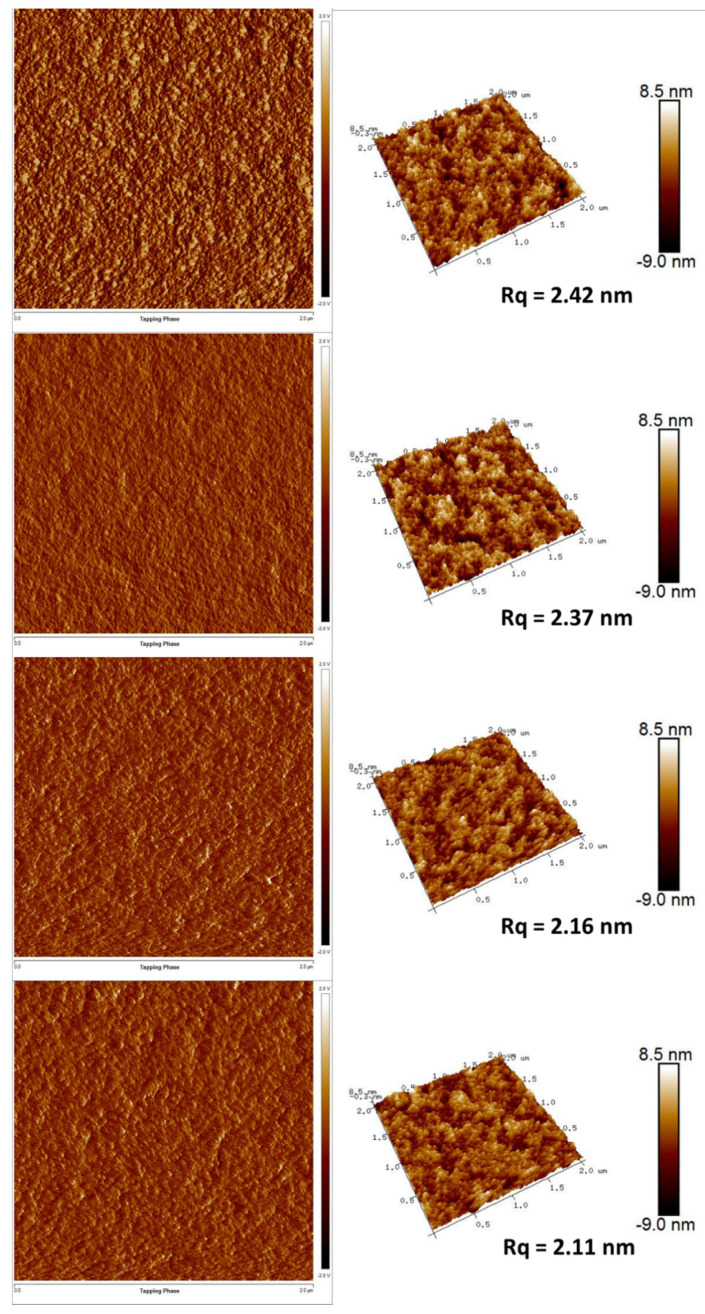
AFM phase (**left**) and topography (**right**) images for VB-FNPD films prepared by different standing times (top to bottom: W/O standing, 20, 40 and 60 min).

To further improve the device performance, the different standing times were introduced into the VB-FNPD film preparation. Figure 4 shows current–voltage–luminance characteristics, EQE and the normalized electroluminescence (EL) spectrum of the devices prepared by different standing times. The performances of all devices with the standing treatment show better than that of the device without the treatment. EL spectrum shows an emission peak at 680 nm with a narrow FWHM of 32 nm, indicating high color purity. The EL peak position is close to the PL spectrum, which can be attributed to carrier recombination in the QD films. Figure 5 shows the AFM images of the VB-FNPD films with the different standing times. The AFM phase image exhibits that light and dark colors are alternately and uniformly distributed on the VB-FNPD film surface without the standing treatment, indicating two-phase coexistence [33] and low degree of polymerization. With the increase in the standing times, the deepened colors and the larger domain sizes on the phase images can be found, which could be attributed to the increased degree of the polymerization. Therefore, the reduced surface roughness can be seen in the AFM topography images, leading to the improved hole transporting characteristic and the device performance. Thus, the highest EQE of 8.64% in the VB-FNPD devices treated for 60 min were achieved.

## 4. Conclusions

In conclusion, polymeric hole transport materials employed for red CsPbI_3_ IPQLEDs have been demonstrated. The band-aligned and aggregation characteristics of the CsPbI_3_ layers deposited on P3HT, PVK, Poly-TPD and VB-FNPD HTLs were discussed. A full-coverage QD film without the aggregation can be obtained on the VB-FNPD films, and thus, the best performance was 7.28% in the VB-FNPD device. One of the key issues associated with the utilization of thermal-crosslinking polymer thin films is the control of their alignment and orientation. A standing method of increasing the degree of VB-FNPD polymerization was also presented, resulting in the improved device performance with the EQE up to 8.64%.

## Figures and Tables

**Table 1:** Summary of recent reports of CsPbI_3_-based all-inorganic perovskite quantum-dot light-emitting diodes (IPQLEDs).

Years	Emission Layer	EL Wavelength (nm)	HTLs	Peak EQE (%)	Maximal LUMINANCE (cd/m^2^)	Reference
2017	CsPbI3	688	Poly-TPD	5.02	748	[15]
2018	CsPbI3	694	Poly-TPD	14.08	1444	[16]
2019	CsPbI3	682	Poly-TPD	1.8	365	[17]
2020	CsPbI3	687	PTAA	14.6	378	[18]
2020	CsPbI3	676	Poly-TPD	6.2	3762	[19]
2020	CsPbI3	675	Poly-TPD	10.21	401	[20]
2020	CsPbI3	685	Poly-TPD	6.02	587	[21]
-	CsPbI3	680	VB-FNPD	8.64	632	This work

**Table 2:** Summary of photoluminescence quantum yield (PLQY) values of CsPbI_3_ quantum dot (QD) films coated on a glass and different hole transporting layers (HTLs).

	Glass	VB-FNPD	Poly-TPD	PVK	P3HT
PLQY (%)	46.7	42.6	18.0	17.5	15.3

## Data Availability

Statement excluded.

## Supplementary Materials

The following are available online at https://www.mdpi.com/2073-4360/13/6/896/s1, Figure S1: Impedance characteristics of IPQLED devices with different HTLs.
