# Supplementary material for: Polymeric Hole Transport Materials for Red CsPbI3 Perovskite Quantum-Dot Light-Emitting Diodes
Source: Polymers (Basel). 2021 Mar 15;13(6):896. doi: 10.3390/polym13060896 (PMC7999490; doi:10.3390/polym13060896)
Supplement: Supplementary file 1 [file polymers-13-00896-s001.pdf]

Supporting information

Polymeric Hole Transport Materials for Red $CsPbI_3$ Perovskite Quantum-dot Light-emitting Diodes

Zong-Liang Tseng[1*], Shih-Hung Lin[2], Jian-Fu Tang[3], Yu-Ching Huang[4], Hsiang-Chih Cheng[5], Wei-Lun Huang[1], Yi-Ting Lee[6] and Lung-Chien Chen[5*]

[1]Department of Electronic Engineering and [4]Department of Materials Engineering, Ming Chi University of Technology, No. 84, Gungjuan Rd., New Taipei City 24301, Taiwan.

[2]Department of Electronic Engineering and [3]Bachelor Program in Interdisciplinary Studies, National Yunlin University of Science and Technology, Yunlin 64002, Taiwan.

[5]Department of Electro-optical Engineering, National Taipei University of Technology, 1, Sec.3, Chung-Hsiao E. Rd., Taipei 106, Taiwan.

[6]Center for Organic Photonics and Electronics Research (OPERA) Kyushu University 744 Motooka, Nishi, Fukuoka 819-0395, Japan.

*Corresponding authors. E-mail:

zltseng@mail.mcut.edu.tw (Z. L. Tseng)

ocean@ntut.edu.tw (L. C. Chen)

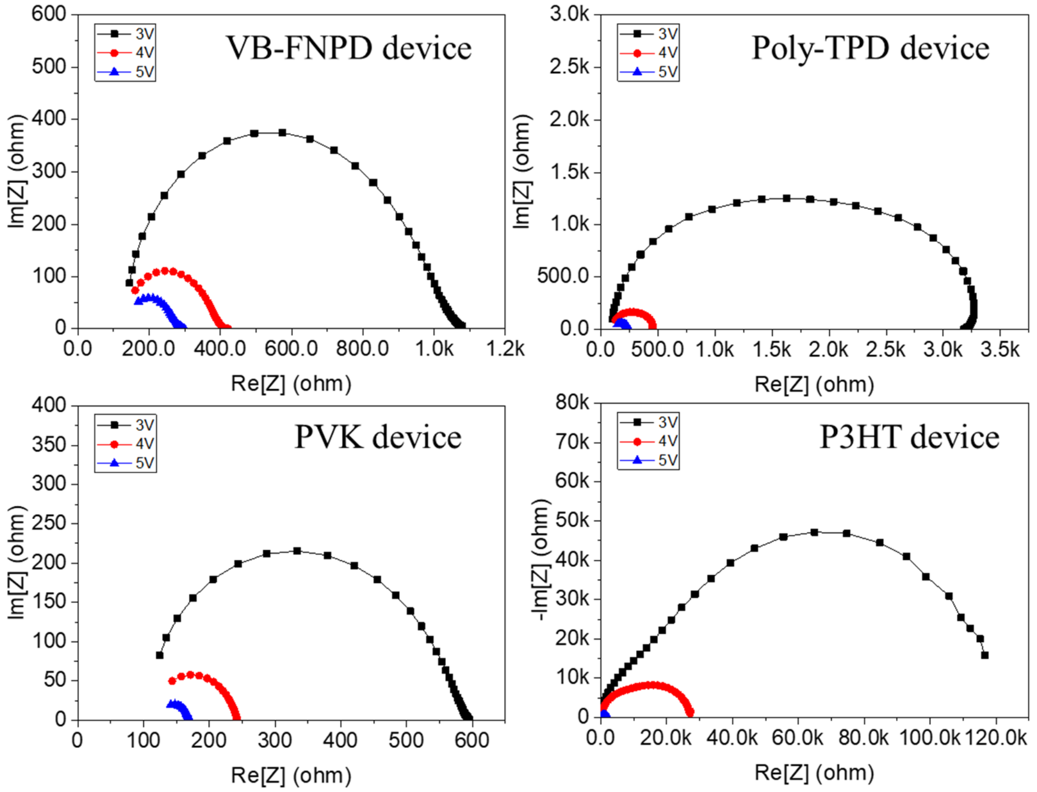

Figure S1 Impedance characteristics of IPQLED devices with different HTLs.
